# Sustainable cereal production: A spatial analytical approach using the Ghana living standards survey

**DOI:** 10.1016/j.heliyon.2023.e17831

**Published:** 2023-07-03

**Authors:** Daniel Adu Ankrah, Nana Afranaa Kwapong, Seth Awuku Manteaw, Fred Fosu Agyarko

**Affiliations:** a University of Ghana, Department of Agricultural Extension, School of Agriculture, College of Basic and Applied Science (CBAS), P. O. Box LG 68, Legon, Accra, Ghana; b Council for Scientific and Industrial Research (CSIR) - Institute for Scientific and Technological Information (INSTI), P. O. Box M32, Accra, Ghana

**Keywords:** Maize, Farming households, Male-headed households, Female-headed households, Agriculture

## Abstract

Ghana as one of the countries south of the Sahara, depends solely on cereals as a major staple food. Ironically, Ghana's economy depends on large importation from the global north, particularly Asia, due to systemic production deficits. The probability of farming households producing enough cereals and the constraints to meeting domestic supply remains imperative. Therefore, the current research focussed on the Ghana Living Standard Survey seventh round (GLSS7) involving 15,045 cereal farmers nationwide. By estimating the probability of farming households producing cereals and the factors that constrain cereal production. Using random-effects regression models, the empirics show that farming households are expected to produce 5.87 tonnes of cereals annually. Specifically, farming households headed by males are expected to produce 6.01 tonnes of cereal crops in a year, 0.14 tonnes more than female-headed households. Non-poor households are expected to produce 6.82 tonnes of cereals in a year compared with an expected production of 6.29 tonnes by poor households. Cereal production is constrained by wealth status, gender, and age of household heads. Our findings attempt to inform and shape policy towards sustained cereal production in Ghana, and by implication countries in sub-Saharan Africa (SSA). The Ministry of Food and Agriculture (MoFA) in Ghana, should bring on board a structural policy that will address constraints related to gender, wealth, and age of household heads to enhance sustainable cereal production.

## 1 Introduction

Currently, high dependence on cereals importation into Ghana, and by implication, most countries in sub-Saharan Africa (SSA) is a serious threat to sustainable food production in the region. For instance, Ankrah, Agyei-Holmes [1] indicated that the mere mention of rice resonates with foreign rice in a critical mass of Ghanaian households, pointing to the high dependence on cereal imports. Specifically, in 2020, cereals imported to Ghana were estimated around 2.13 million tonnes. Cereals imports increased from 77,045 tonnes to 2.13 million tonnes in 1971 and 2020 respectively, at an average annual growth rate of 13.03% [2]. In an attempt to understand the heavy dependence on cereal importation, the related extant literature abounds on the factors that constrain cereal production, but with rare evidence on the probability of farmers producing cereals using a nationally representative dataset. Specifically, this research therefore, interrogates the probability of a farmer producing cereals by spatial location and the factors that constrain the production using the Ghana Living Standard Survey seventh round (GLSS7).

The essence of food and nutrition cannot be taken for granted, as rightly reflected in the United Nations (UN's) 2030 Agenda for Sustainable Development through the 17 Sustainable Development Goals (SDGs), with particular mention of goal 2 which seeks to end hunger and promote sustainable agriculture [3]. The Comprehensive Africa Agriculture Development Programme (CAADP) designed by the African Union (AU) strongly focuses on sustainability with emphasis on diet diversity as a critical outcome [4].

The production of cereals including sorghum, millet, maize, and rice are important to ensure Ghana's food sovereignty and that of other SSA countries in the food security campaign in the region. Agriculture in Ghana is the most important economic sector employing more than half the population in both formal and informal sectors, and providing food and export products and approximately half of the gross domestic product (GDP) [5]. This indicates that if the nation can produce more tonnes of cereal crops, the country may improve on its GDP and also address any possible food insecurity issues.

However, spanning several decades, there has been low production of cereals and this pattern has been a challenge for most countries in SSA [3]. Challenges accounting for the low cereal production include issues with climate crisis, land degradation, conflict, low use of fertiliser (SSA's average use is 11 kg/ha relative to the world average of 62 kg/ha), limited access to markets, limited use of improved seed, low level of knowledge, food losses due to pests and diseases in the field, high post-harvest losses, high poverty levels, policy orientation, low investment in mechanisation, high population rate (3% annually) that is not at par with the rate of food production, trade imbalances, low use of technology, and lately economic shocks due to COVID-19 [1,[6], [7], [8]].

Aside from the known challenges relating to the production of cereals, there continues to be a dearth of literature about the probability of producing cereals in Ghana by spatial location. The extant literature [[9], [10], [11], [12]] analysing factors hindering cereal crops production in Ghana rarely involves count data estimation techniques. For instance, the most recent study on cereal crops mitigating the food insecurity menace on the African continent [3] employed documentary analysis. The need to employ rigorous estimation approaches that can predict the probability of producing or not producing cereals using statistical approaches remains essential to assess the future of cereals and the country's overall cereal food (in)-security. This research is animated by this concern. Specifically, we find that most datasets in the agricultural sector (agriculture produce) are basically count data. For instance, cereal crops are measured in tonnes; in such a case, the Poisson regression model is the best estimation approach for fitting such a dataset. Although, the Poisson regression fits count data perfectly, it becomes incapacitated when the count data contain excess zeros. In the agricultural sector, this phenomenon is very common with the number of tonnes of a particular crop (agriculture produce) produced by farmers in a locality in a specific period of time [13]. This violates the fundamental assumption (the expectation and the variance must be the same under this distribution) of the Poisson regression model; thus, it creates overdispersion in the model. This implies that the variance may be greater than the expectation. The Negative Binomial remedies the situation (i.e., the Negative Binomial regression takes care of overdispersion in models). Since there are excess zeros in the dataset, the Poisson and the Negative Binomial regression models have been extended to accommodate the zeros. This gives rise to the zero-inflated models (i.e., the case of Zero-Inflated Poisson (ZIP) and Zero-Inflated Negative Binomial (ZINB) models).

Generally, this study seeks to investigate the socioeconomic, spatial, and demographic factors assumed to be potential constraints to cereal production in Ghana using hierarchical zero-inflated regression models. Given the low production of cereals, the study seeks to estimate the probability of a farming household to produce cereals based on spatial location. The paper's two objectives appear to be novel, given that to the best of our knowledge, no study in Ghana has attempted to undertake such analysis, even though it is useful for formulating policy in addressing Ghana's over reliance on cereal importation.

## 2 Materials and methods

### 2.1 Sources of data and variables in the model

The study's source of data is the Seventh Round of the Ghana Living Standard Surveys (GLSS 7) implemented by the Ghana Statistical Service (GSS). The data covers all the16 administrative regions in Ghana (viz, Ashanti, Brong Ahafo, Central, Eastern, Greater Accra, Northern, North East, Savannah, Upper East, Upper West, Western, Western North, and Oti, Volta regions). Note however that, at the time the survey was conducted, Ghana had 10 administrative regions. The regions in northern Ghana were divided to give additional two regions i.e. Savannah Region, and Nort East Region. Volta Region was also divided to give a new region Oti Region, the Brong Ahafo Region was also divided to give Ahafo and Bono East regions. The Western Region was divided to give the Western North Region. The data constitute a nationally representative household survey conducted over 12 months [14]. The GLSS 7 is the seventh comprehensive multipurpose household survey that evaluates Ghanaians living conditions.

The farming households that were captured during the period of the survey were 15,045 in total. We had no missing cases in the dataset, implying that the analysis made in this study on factors influencing the number of tonnes of cereals produced by a farming household was conducted on all farming households (15,045) in Ghana. The response variable for the study was the number of tonnes of cereals produced in a year by a farming household. The study's covariates were some related socioeconomic, spatial, and demographic factors that are assumed to be potential hindrance to the production of cereals in Ghana.

### 2.2 Statistical methods

#### 2.2.1 Background

In this study, random-effects regression models are employed for analysing our dataset which is a clustered data. Different from the classical regression analysis of clustered data, which assume independent observations, the random-effects regression models assume that observations are not independent of each other and assume that there is some degree of dependence among data within clusters. In view of this, the researchers expect to extend the ZIP and ZINB models to accommodate sources of heterogeneity triggering the “over dispersion phenomenon.” With regard to the study data (clustered data), it is prudent to accommodate this extension to correct for hierarchical structures and also account for potential correlation within outcomes from the same subject.

Many studies have extended the Poisson and the Negative binomial distributions to accommodate excess zeros. In a much earlier study, Lambert [15] extended the Poisson distribution to accommodate excess zeros by combining a Poisson distribution and a discrete mass to obtain a zero-inflated model. The Poisson part generates both zeros and counts, while the discrete mass part (typically binomial distribution) also generates only zeros. However, the ZIP model becomes inappropriate when the count part of the distribution is over-dispersed. Therefore, it is prudent to substitute the Poisson part by a negative binomial distribution to fit the data and this results in the birth of zero-inflated negative binomial (ZINB) distribution [16]. The negative binomial aspect, assumes that the Poisson mean is a random variable that follows a gamma distribution.

There have been many studies on ZI models that have accommodated random-effects. There has also been extensive work on zero-inflated models. Hall [17], much earlier, adapted Lambert's methodology to an upper bounded count situation (zero-inflated binomial model) and added the flexibility of the fixed effects models by introducing random effects. Lee, Wang [18] also employed an independent random effect for the Poisson and the binary mixture components.

Fang, Wagner [19] applied the zero-inflated negative binomial mixed model on two microbial organisms important in oesophagitis. Also, Zhu, Luo [20] extended the zero-inflated mixed models to account for random effects heterogeneity by modelling their variance as a function of independent variables. They established through simulation that ignoring intervention and covariate-specific heterogeneity can produce biased estimates of covariate and random effect estimates.

Similarly, Xie, Lin [21] examined the corresponding test statistic, interrogated the sampling distribution and power of the score test statistic via Monte Carlo simulation. Recently, Ghasemi, Akbarzadeh Baghban [22] introduced double-inflated Poisson models for zero-inflated and count-inflated data. The study aims to introduce a Doubly-Inflated Poisson models with random effect for correlated doubly-inflated data.

#### 2.2.2 POISSON GLMM

Given that $Y_{i}$ represents the number of tonnes of cereal crops produced by farming households i=1,2,3,...,n; then with regard the Poisson regression, $Y_{i}$ follows a Poisson distribution with an expectation, $\mu_{i}$. The expectation has a relation with the set of p- covariates, , through a log-link function. Thus, $E\left(Y_{i}\right)=\mu_{i}=\exp\left(\beta^{\prime }X\right)$, where β is the effect of the covariates. Under this distribution, the variance has the same value as the expectation. Thus, variance of $Y_{i}$ is also $\mu_{i}$. The Poisson distribution becomes less efficient when the variance is either higher or lower than the expectation. When this phenomenon happens, it is termed either overdispersion or under dispersion.

A key assumption of the Poisson distribution is the independence assumption of observations. However, in situations where count data observed are clustered or repeated over time, the independence assumption is violated. Thus, observations in individual clusters will be highly correlated. This can be addressed by introducing random-effects in the linear part of the relationship linking the marginal means. For instance, with regard to the Poisson model, we have.

$\log\left(\mu_{ij}\right)=\beta 'X+Z'b\;\log\left(\mu_{ij}\right)=\beta 'X+Z'b$, where $\mu_{ij}$ is the marginal mean of subject, i, at time j or cluster j, b∼N(0,D) is a vector of random effects, and Z is a set of predictors associated with the random effects.

The likelihood of the model is obtained by integrating the random effects. That is, $L=\int \prod_{i=1}^{n}f\left(Y|b_{i}\right)db_{i}$. For the Poisson mixed-effects model with random intercepts only, the marginal mean and variance are, respectively captured in equations (1), (2)) below:

(1) $$E\left(Y_{i}\right)=\mu_{i}=\exp\left(\beta^{\prime }X+\frac{d^{2}}{2}\right)$$

(2) $$var\left(Y_{i}\right)=\mu_{i}\left(1+\mu_{i}\left(\exp\left(d^{2}\right)-1\right)\right)$$

where $d^{2}$ is the variance of the random intercept. It can be noticed from the equations the generalised linear mixed models account for the overdispersion through the parameter, d*.*

### 2.3 NBGLMM

The negative binomial model also known as Poisson-gamma is an extension of the Poisson model. This model addresses the problem of over dispersion that the Poisson is limited in addressing. A Poisson model extension addresses possible over-dispersion in data. The negative binomial model assumes that the Poisson parameter takes on a gamma probability distribution. With this analogy, the PMM can be transformed into the NBMM to mitigate the problem of overdispersion by assuming gamma distribution errors.

Suppose that X and Z are known vectors of covariates associated with count data $Y_{i}$, i=1,2,3,...,n at cluster j count on a p dimensional vector of subject-specific random effects, $b_{i}$. Hence with the gamma errors assumption $Y_{i}$ has a negative binomial distribution, $NB∼\left(\mu_{ij},\mu_{ij}+\eta \mu_{ij}^{2}\right)$. For the negative binomial mixed-effects model with random intercepts only, the marginal mean and variance are, respectively captured in equations (3), (4)) below:

(3) $$E\left(Y_{i}\right)=\exp\left(\beta^{\prime }X+\frac{d^{2}}{2}\right)$$

(4) $$var\left(Y_{i}\right)=\exp\left(\beta^{\prime }X+\frac{d^{2}}{2}\right)+\eta {\left[\exp\left(\beta^{\prime }X+\frac{d^{2}}{2}\right)\right]}^{2}$$

The problem of overdispersion and correlation among observations can both occur in a model. In remedying these problems, Molenberghs, Verbeke [23] developed a flexible and unified modelling framework (the combined model) that concurrently detects overdispersion and association in clustered data and longitudinal.

### 2.4 ZIP GLMM

In reality, most count data contain many zeros; and this phenomenon is not alien to agricultural dataset. It is very possible that farmers may not produce a certain type of crop in specified time period due to factors such as lack of finance, bad weather, natural disaster etc. This makes the classical model less efficient in fitting such data. In such situations, data are fitted as a zero-inflated model. The ZI model splits the model into two, the positive counts (with probability $1-\eta_{i}$) against zero counts (with probability, $\eta_{i}$). For a zero-inflated Poisson generalised linear model (ZIP) the probability density function is given by equation (5) below:

(5) $$P\left(Y_{i}=y_{i}\right)=\left\{ \begin{array}{l} \left(1-\eta_{i}\right)\frac{\exp\left(-\lambda_{i}\right)\lambda_{i}^{y_{i}}}{y_{i}!}\;\text{when}\;y_{i}>0\\ \eta_{i}+\left(1-\eta_{i}\right)\exp\left(-\lambda_{i}\right)\;\text{when}\;y_{i}=0 \end{array}\right.$$

Since the ZIP model does capture correlation among observations, the ZIP model is extended to the zero-inflated Poisson generalised mixed model accommodate the correlation among observations (i.e., the ZIPOISSON GLMM corrects for the dependency in the observations). This indicates that random-effects are introduced in ZIP model. The expectation and variance of the ZIPOISSON GLMM with random intercepts only are defined respectively by equations (6), (7)) below:

(6) $$E\left(Y_{i}\right)=\exp\left(\beta^{\prime }X+\frac{d^{2}}{2}\right){\left[1+\exp\left(\alpha^{\prime }Z+\frac{d^{2}}{2}\right)\right]}^{-1}$$

(7) $$var\left(Y_{i}\right)=\exp\left(\beta^{\prime }X+\frac{d^{2}}{2}\right)\left[1+\exp\left(\beta 'X+\alpha 'Z+d^{2}\right)\right]$$

where X, Z are vectors of parameters with α and β, respectively, and $d^{2}$ is the variance of the random intercept.

### 2.5 ZINB GLMM

Zero-Inflated Negative Binomial (ZINB) regression model is similar to the ZIP distribution, it is also a mixture of distributions. The ZINB is also two separate data generation processes, the positive counts (with probability $1-\eta_{i}$) and the zero counts (with probability, $\eta_{i}$) is governed by a negative binomial with an expectation λ. The probability of zero counts can be generated by combining probability of zeros from the two processes. Thus, the probability distribution of the ZINB is given by equation (8) below:

(8) $$P\left(Y_{i}=y_{i}\right)=\left\{ \begin{array}{l} \left(1-\eta_{i}\right)\frac{\Gamma \left(y_{i}+\tau \right)}{y_{i}!\Gamma \left(\tau \right)}{\left(1+\frac{\lambda_{i}}{\tau }\right)}^{-r}\left(1+\frac{\lambda_{i}}{\tau }\right)\;\text{when}\;y_{i}>0\\ \eta_{i}+\left(1-\eta_{i}\right){\left(1+\frac{\lambda_{i}}{\tau }\right)}^{-r}\;\text{when}\;y_{i}=0 \end{array}\right.$$

Moghimbeigi, Eshraghian [24] formulated multi-level ZINB regression to mitigate the problems of zero-inflation and correlation may occur simultaneously in a model. Given $\mu_{ij}$ is the marginal mean of subject, i, at time j or cluster j, b∼N(0,D) is a vector of random effects, and Z is a set of predictors associated with the random effects. The expectation and the variance of ZINB generalised mixed model are defined by equations (9), (10)) below:

(9) $$E\left(Y_{i}\right)=\exp\left(\beta^{\prime }X+\frac{d^{2}}{2}\right){\left[1+\exp\left(\alpha^{\prime }Z+\frac{d^{2}}{2}\right)\right]}^{-1}$$

(10) $$var\left(Y_{i}\right)=\exp\left(\beta^{\prime }X+\frac{d^{2}}{2}\right)+\eta {\left[\exp\left(\beta^{\prime }X+\frac{d^{2}}{2}\right)\right]}^{2}\left[1+\exp\left(\beta 'X+\alpha 'Z+d^{2}\right)\right]$$

where X, Z are vectors of parameters with α and β, respectively, and $d^{2}$ is the variance of the random intercept.

## 3 Results and discussions

### 3.1 Exploratory analysis

Table 1 describes the independent variables (i.e., socioeconomic, demographic, and spatial factors) employed in this study. The results show that the Greater Accra Region recorded the least number of farming households in Ghana, representing (0.96%). This is unsurprising since Greater Accra is Ghana's capital city characterised by industrial and non-agricultural activities. Due to this, the region has become the business and commercial hub of the country. Most of the lands have been converted to industrial, commercial, business and residential edifices [25]. The three northern regions^1^recorded the highest number of farming households; the Northern, Upper West and Upper East regions recorded 19.79%, 16.96% and 16.70% respectively. The middle age category (36–60) recorded the highest (56.70%) in terms of household heads, followed by those above 60 years (22.17%) and the youth category recorded the least percentage (21.13%). Table 1 further shows that about 38.60% of household heads had never been educated formally (i.e., who had not sat in a formal classroom in their entire life), whereas about 61.40% of household heads have at least stepped in a formal classroom before. The gender distribution of household heads was not normal; approximately 76.76% of household heads were male whereas the remaining 23.24% of household heads were females. It can also be seen that most farming households are located in the rural areas of Ghana. Majority (90.70%) of farming households were rural residents, whereas a few of the farming households were located in the urban areas in Ghana. It can also be seen that most of the farming households are not poor. About 44.80% of farming households were able to consume more than GH¢1314^2^/US$ 298.63 per year on food and non-food commodities. Whereas those who were classified as very poor (food and non-food consumption of GH¢792.05/US$ 180.01 per year) constituted about 27.6% of farming households in Ghana.

**Table 1:** Descriptive statistics of the covariates.

Variable	Category	Frequency	Percent (%)
Region	Greater Accra	144	0.96
	Western	1024	6.81
	Central	960	6.38
	Eastern	1206	8.02
	Volta	1488	9.89
	Ashanti	677	4.5
	Brong Ahafo	1505	10
	Northern	2977	19.79
	Upper East	2552	16.96
	Upper West	2512	16.7
Age	15–35	3179	21.13
	36–60	8530	56.7
	Above 60	3336	22.17
Gender	Male	11549	76.76
	Female	3496	23.24
Education	Never	5814	38.64
	Educated	9231	61.36
Residence	Urban	1400	9.31
	Rural	13645	90.69
Wealth status	Very poor	4147	27.56
	Poor	4158	27.64
	Non-poor	6740	44.8

**Fig. 1:**
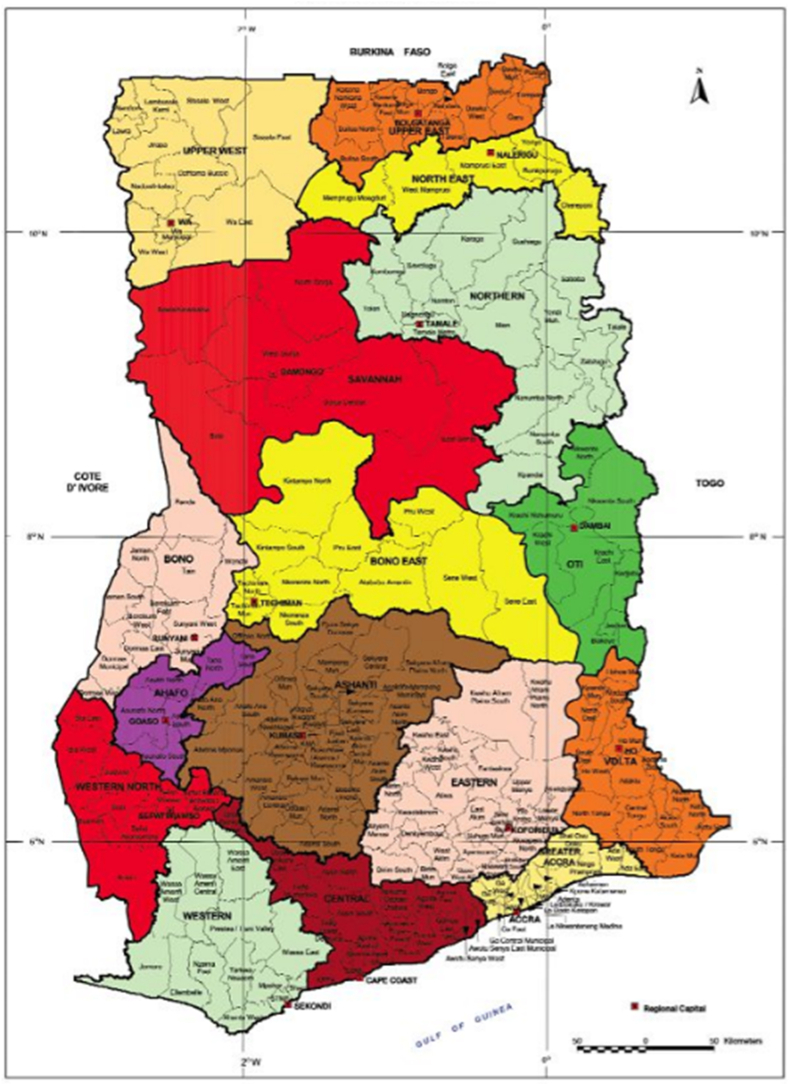
Administrative map of Ghana showing the Sixteen Regions of Sustainable Cereals Production. Source: Ghana Statistical Service, Geographical Information System (GIS) Section.

Footnote: Please note that the regions in northern Ghana were divided to give additional two (2) regions i.e. Savannah Region, and Nort East Region. Volta Region was also divided to give a new region Oti Region, the Brong Ahafo Region was divided to give Ahafo and Bono East regions. The Western Region was divided to give the Western North Region (see Fig. 1).

### 3.2 Estimation of coefficients

**Fig. 2:**
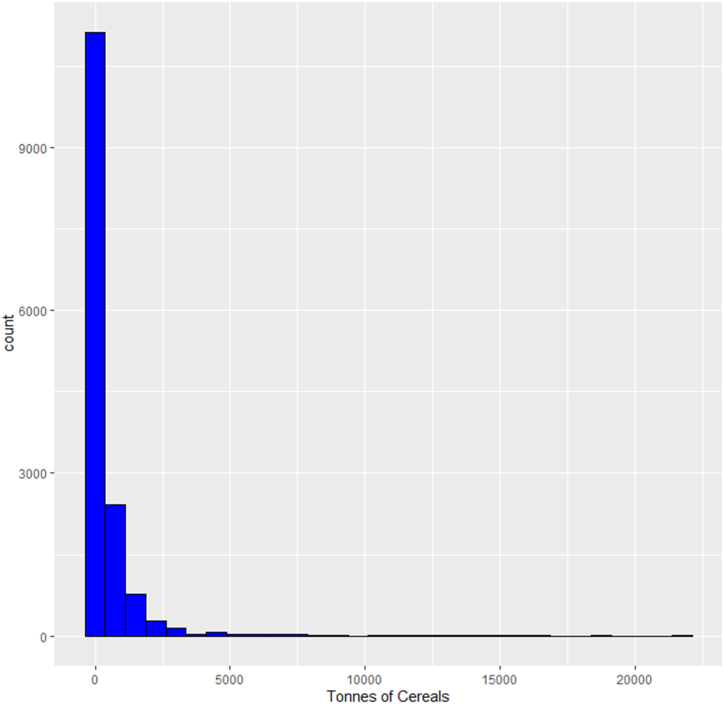
Number of tonnes of cereals produced by farming households in Ghana.

Employing the overdispersion test in the Applied Econometrics in R (AER) package in R statistical software indicates the presence of overdispersion in the Poisson regression model. The results, show that the mean and variance of the response variable (number of tonnes of cereals produced by farming households in a year) confirms the presence of overdispersion. Thus, the variance (1975682) is much greater than the mean (446.73), indicating a violation in the underlying assumption of the Poisson distribution (the mean is equal to the variance). Furthermore, the distribution of the dependent variable has (tonnes of cereals produced) a large proportion of zeros; this is evident in Fig. 2 and Table 2.

**Table 2:** Number of tonnes of cereals produced by farming households in Ghana (Pooled).

Number of tonnes	Frequency	Percent (%)
0	9739	64.732
11	1	0.013
14	1	0.006
16	1	0.006
18	3	0.02
24	6	0.04
…	… (Omitted)	…
15126	5	0.033
15418	7	0.047
16819	3	0.02
18922	5	0.033
21725	7	0.047

The study employed Akaike's Information Criterion (AIC) and Bayesian Information Criterion (BIC) as performance measures for all models used. These performance measures (model selection criteria) were used because of their unique abilities to penalise models for adding irrelevant parameters. It could be seen in Table 3 that the Poisson generalised linear mixed model recorded the highest values in AIC and BIC; this indicates that it is the poorly performed model among the four models used. It can be noticed that the Zero Inflated Negative Binomial generalised linear mixed model (ZINBG) was the performing model in terms of Both AIC and BIC. Against an apriori expectation, the Negative Binomial generalised linear mixed model (NBG) outperformed the Zero Inflated Poisson generalised linear mixed model (ZIPOISSON GLMM). This indicates that the NBG may have corrected for excess zeroes compared to the ZIPOISSON GLMM.

**Table 3:** Performance measure for models.

Models	Degree of Freedom	AIC	BIC
POISSON GLMM	9	15656266.4	15656335
NB GLMM	10	111306	111382.1
ZIP GLMM	18	6210652.7	6210789.9
ZINB GLMM	19	101816.2	101960.9

As earlier indicated, four generalised linear mixed models were fitted to the data. Table 4 presents the results from the analysis. In all four models, the number of tonnes of cereal produced by farming households in a year was in terms of the gender of the household head, age category of household heads wealth index of the household, the educational level of the household head, and also nested households within regions and residential location.

**Table 4:** Estimation of coefficients for positive counts using different count data models for number of tonnes of Cereals.

Variables	POISSON		NB		ZIP		ZINB	
	Estimate	Std. Error	Estimate	Std. Error	Estimate	Std. Error	Estimate	Std. Error
(Intercept)	3.269***	0.410	3.690***	0.417	5.486***	0.161	5.866***	0.150
**Gender**								
Female	1	–	1	–	1	–	1	–
Male	0.430***	0.001	0.333***	0.084	0.295***	0.001	0.137***	0.033
**Education**								
No	1	–	1	–	1	–	1	–
Yes	−0.114***	0.001	−0.025	0.074	−0.130	0.001	−0.050	0.027
**Wealth status**								
Very Poor	1	–	1	–	1	–	1	–
Poor	0.755***	0.001	0.777***	0.097	0.440***	0.001	0.424***	0.034
Non-poor	1.611***	0.001	1.158***	0.099	1.175***	0.001	0.948***	0.036
**Age category**								
Youth	1	–	1	–	1	–	1	–
Middle	0.382***	0.001	0.331***	0.087	0.302***	0.001	0.216***	0.033
Aged	0.449***	0.001	0.279***	0.104	0.298***	0.001	0.218***	0.039
**Random-effects**								
	**Variance**		**Variance**		**Variance**		**Variance**	
Region	1.588	–	1.336	–	0.253	–	0.187	–
Residence	0.019	–	0.036	–	0.0088	–	0.0035	–

***P < 0.001, **P < 0.01, *P < 0.05, Std. Error. = Standard error.

**Fig. 3:**
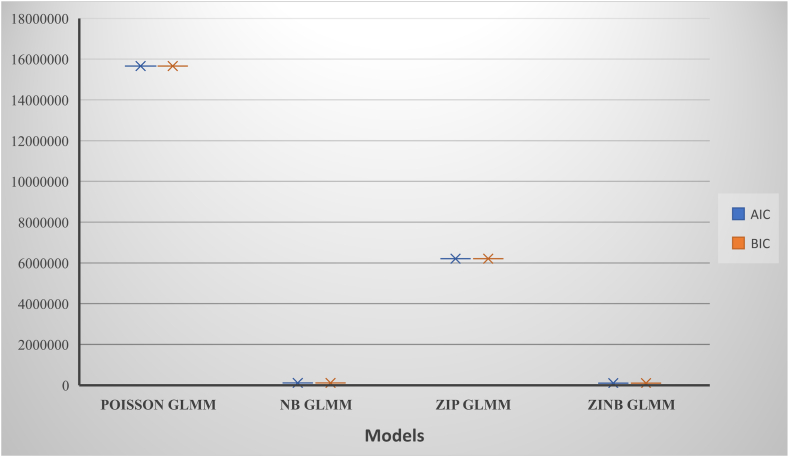
Performance measure for models.

With regard to the count models, it can be noticed that all the covariates in the Poisson models (i.e., POISSON GLMM and ZIP GLMM) were all significant. On the other hand, all the covariates in the Negative Binomial models (NB GLMM and ZINB GLMM) were significant, with the exception of educational status (Please see Table 4 & Fig. 3).

Since ZINB GLMM was the best performing model among the models employed, we target interpreting the results associated with it. Table 4 shows that the model had an intercept estimate of 5.87 tonnes of cereal crops (See Fig. 3). This indicates that when there are no socio-economic, demographic, and geographical factors present (i.e., when all covariates are set to zero), the expected number of tonnes of cereal crops produced in a year by a farming household in Ghana is approximately 5.87 tonnes. The results show that the demographic factor “*gender*” was significant at 5%. This factor was a dummy variable (Male and Female) with the category “*Female*” set as the baseline. The “*Male*” category recorded an estimate of 0.14. This implies that holding all covariates constant, a farming household headed by a male is expected to produce 6.01 tonnes of cereal crops in a year (i.e., 0.14 more of what a household headed by a female is expected to produce in a year). Indeed, the literature [[26], [27], [28]] shows that male-headed households tend to produce more than female-headed households in Ghana. Codjoe [29] provided a contrary finding that showed that female-headed households produced more than male-headed households for maize production in Ghana.

The next significant variable is a socio-economic variable “*wealth status*”, which was significant at 0.1%. It is a categorical variable, as earlier indicated. The variable was categorised into *very poor*, *poor* and *non-poor*. In this case, the *very poor* category was used as the baseline. It is important to note that *poor* recorded 0.42, indicating that all other things being equal, a farming household that is regarded as poor (households that consume above GH¢ 792.05 - GH¢1314 per year on food and non-food commodities) is expected to produce 6.29 tonnes of cereal crops in a year. Thus, a poor farming household is expected to produce 0.42 tonnes more than a very poor farming household. It can be seen that the category *non-poor* (households that consume above GH¢1314 per year on food and non-food commodities) recorded an estimate of 0.95. This also indicates that all other things being equal, a non-poor farming household is expected to produce 6.82 tonnes of cereal crops in a year as compared to a very poor farming household. Consequently, non-poor households are expected to produce approximately 0.95 tonnes and 0.53 tonnes of cereal crops in a year than very poor and poor households respectively.

The results show that the age category “*aged”* (i.e., household heads who are more than 60 years) was also very significant. The age category *middle* (i.e., household heads that are between 36 and 60 years) was also significant at 0.1%. The age category *youth* was set as the baseline. It can be seen that the *middle* category recorded an estimate of 0.22. This implies that holding all covariates constant, a farming household headed by a middle-aged person is expected to produce 6.09 tonnes of cereal crops in a year as compared with a farming household headed by a youth.

The last two rows in Table .5 also provide the random-effects component in the models. Our focus will be on the ZINB GLMM model since it was the best performing model among the others. It can be seen that the count model recorded a residual variance, regional variance and residence variance of 0.1233, 0.1865 and 0.0003 respectively. For farming households within regions, the model recorded an intraclass correlation (f = al/[a2 + al]) of 0.602. This indicates that the clustering of farming households within regions accounted for approximately 60.2% of the variability in the dataset that is not explained by covariates in the model. Similarly, the intraclass correlation for the number of tonnes of cereal crops produced by farming households within residence was 0.0027. This also implies that there is about 0.27% of total unexplained variability in the dataset. In summary, there was more homogeneity in the number of tonnes of cereal crops produced by farming households when clustered by regions as compared to clustering by residence.

**Table 5:** Estimation of coefficients for zero counts using ZINB and ZIP models for number of tonnes of Cereals.

Variables	ZIP GLMM		ZINB GLMM	
	Estimate	Std. Error	Estimate	Std. Error
(Intercept)	2.329***	0.416	2.328***	0.416
**Gender**				
Female	1	–	1	–
Male	−0.211	0.046	−0.211	0.046
**Education**				
No	1	–	1	–
Yes	−0.044	0.040	−0.044	0.040
**Wealth status**				
Very poor	1	–	1	–
Poor	−0.610***	0.049	−0.610***	0.049
Non-poor	−0.716***	0.051	−0.715***	0.051
**Age category**				
Youth	1	–	1	–
Middle	−0.204***	0.048	−0.204***	0.048
Aged	−0.239***	0.057	−0.239***	0.057
**Random-effects**				
	**VARIANCE**		**VARIANCE**	
Region	1.387	–	1.3872	–
Residence	0.0524	–	0.0524	–

Regarding the zero-inflated model, the intercept recorded an estimate of 4.437. This implies that holding all covariates to zero, a farming household is expected to produce an odds ratio of 84.52. This means that with the absence of these socio-economic, demographic, and geographical factors a farming household is likely to produce approximately 84.52% less of the number of tonnes of cereal crops produced in a year (Please see Table 5).

The education status became significant (as compared to the count model). It is important to note that the education status recorded an estimate of −0.044, thus, farming household heads with formal education are more likely to produce non-zeros in terms of tonnes of cereal crops in a year as compared to farming household heads who have no formal education. The odds ratio is 0.96, this implies that a farming household head that has a formal education is approximately 0.96 times less likely to produce a zero tonne of cereal crops in a year as compared to a household head with no formal education. Whereas the literature [[30], [31], [32]] generally establishes that higher education positively influences the adoption of agricultural innovations, our findings generate new findings on the contrary that when it comes to producing cereals in Ghana, highly educated individuals are less likely to produce maize.

Finance has always been a major factor in Ghana's agricultural sector [33]. It did not come as a shock when poverty status (wealth index) of a farming household was highly significant and its impact on the number of tonnes of cereal a farming household produces is positive. We observe that the odds ratio for a farming household with poverty status as poor is 0.54, this indicates that a farming household with poverty status as poor is 0.54 times likely to produce less zeros of the number of tonnes of cereal as compared to a farming household with poverty status as very poor. Similarly, a farming household with poverty status as non-poor has an odds ratio of 0.49, which indicates that a farming household in this category is 0.49 times less likely to produce more zero tonnes of cereals than a household with poverty status as very poor. Our findings reveal that there is a positive relationship between the wealth status of a farming household and cereal production in Ghana. This is consistent with a study conducted by Appiah-Twumasi, Donkoh [34], where they found that farmers personal savings influences maize production. Several studies [[34], [35], [36]] have established that increased financial capacity of farmers positivity influences agricultural production. Specifically, Anetor, Ogbechie [37] in their study established that commercial loans had a positive effect on agricultural production. Similarly, Agbodji and Johnson [38] found that access to credit had a positive relationship with cereal production.

It is not surprising that the variable “*age category*” was significant in the zero-inflated model and also affected the number of tonnes of cereal crops produced positively. Rationally, household heads who are between the age group “15–35” will have less farming experience as compared to age groups such as “36–60” and “above 60”. It can be noticed that the odds ratio for the age category “*middle*” is 0.82, this implies that a household head who is in his/her middle age is 0.82 times less likely to produce more zeros of the number of tonnes of cereal in a year as compared with a household whose head is youth. It is also less likely for a household head who is aged to produce more zeros as compared to a youth as a household head. The category “*aged*” recorded an odds ratio of 0.79. Essentially the finding points out that the youth are less likely to produce cereals in Ghana. It may be important for the Ministry of Food and Agriculture (MoFA) to entice the youth to venture into agriculture, specifically given that it is a major staple in Ghana. Moreso, most farmers in Ghana are aged [39] and what becomes of the future if the youth are not encouraged to venture into agriculture, leaves sustainable agricultural production in doubt.

The covariate *gender* was also significant at 0.1%. With the female as the baseline, males recorded an odds ratio of 0.81, this indicates that a farming household with a male as the head is 0.81 times less likely to produce more zero tonnes of cereal crops in a year as compared with a household whose head is a female. This points to some gendered participation in maize production that disadvantages female participation. This phenomenon may be partly attributed to unequal access to land, labour and capital [40,41]. The role of gender in the agricultural sector cannot be under emphasised especially in Africa where approximately 70% of the population enjoy their livelihood from the agricultural sector [42]. The findings revealed that gender was statistically significant in cereal production in Ghana. Additionally, the findings show that cereal production increases when the household head is a male relative to when the household is headed by a female.

It can also be noticed that the last two rows in Table 5 provide the random-effects component in the zero-inflated models. As already established, the interpretation of estimates is based on the ZINB GLMM model since it was the best performing model among the others. We observe that the ZI model recorded a residual variance, regional variance, and residence variance of 0.498, 1.387 and 0.0542 respectively. For farming households within regions, the model recorded an intraclass correlation $\left(\hat{r}=\frac{{\hat{\sigma }}_{\alpha }^{2}}{{\hat{\sigma }}^{2}+{\hat{\sigma }}_{\alpha }^{2}}\right)$ of 0.736. This indicates that the clustering of farming households within regions accounted for approximately 73.6% of the variability in the dataset, which is not explained by covariates in the model. Also, the intraclass correlation for the number of tonnes of cereal crops produced by farming households within a residence is 0.095. This also implies that there is about 9.5% of total unexplained variability in the dataset. We summarize that there was more homogeneity in the number of tonnes of cereal crops produced by farming households when clustered by regions as compared to clustering by residence.

## 4 Conclusion

This study estimates the probability of farming households producing cereals and the factors constraining cereal production in Ghana. By exploring the performance of four different mixed linear models on the number of tonnes of cereal crops produced by farming households in all Ghana's sixteen administrative regions. The results demonstrated that among all the count models considered, the Poisson mixed models (POISSON GLMM and ZIP GLMM) performed poorly relative to the Negative binomial mixed models (NB GLMM and ZINB GLMM). This might be due to the fact that the Poisson models tend to be overly restricted. Also, information on the within-subject variability across clusters (i.e., region and residence) was obtained. We found that there was more variability estimated in the ZI part of the model than the count part. The best performing model among the four models considered for this analysis is the ZINB GLMM. It established that all the socio-economic, demographic, and geographic factors employed for the analysis influenced the number of non-zero tonnes of cereal crops produced in a year by a farming household in Ghana.

The nature of the dataset (clustered dataset) paved the way for multilevel models. They were needed because with clustered data, observations within the individual clusters are mostly more similar to each other than the observations across clusters. In such situations, the independence assumption of observations is violated. The intraclass correlation coefficients (a measure of dependence among observations in a cluster) in the study indicate that there is a higher dependency among farming households on a regional basis in both the count and the zero inflated parts of the model compared with residence. There is clear evidence that this kind of analysis must be conducted using random-effects models.

The findings show that farming households are expected to produce 5.87 tonnes of cereals annually. In particular, farming households headed by males are expected to produce 6.01 tonnes of cereal crops in a year, 0.14 tonnes in excess of female headed households. Non-poor households are expected to produce 6.82 tonnes of cereals in a year, compared with an expected production of 6.29 tonnes by poor households. Cereal production is constrained by wealth status, gender, and age of household heads. Our findings attempt to inform and shape policy towards sustained cereal production in Ghana, and by implication, countries in sub-Saharan Africa (SSA). We suggest dedicated attention be paid by the Ministry of Food and Agriculture (MoFA) to addressing structural constraints related to gender, wealth, and age of household heads.

## Limitation of study

This study bulked all cereals (maize, rice, sorghum, millet) together. It might be useful for other studies to disaggregate the different types of cereals to inform specific policy action based on the cereal type.

## Additional information

The data used for this study can be accessed from the Ghana Statistical Service website. With reference ID - DDI-GHA-GSS-GLSS7-2017-v1. https://www2.statsghana.gov.gh/nada/index.php/catlog/97.

## Author contribution statement

Daniel Adu Ankrah; Nana Afranaa Kwapong; Seth Awuku Manteaw; Fred Fosu Agyarko: Conceived and designed the study Performed the analysis; Analyzed and interpreted the data; , analysis tools or data; Wrote the paper.

## Additional information

No additional information is available for this pape.

## Declaration of competing interest

The authors declare that they have no known competing financial interests or personal relationships that could have appeared to influence the work reported in this paper.

## Footnotes

1 Note that due to the creation of new regions, the regions are now five (5) in northern Ghana.

2 Average Ghana cedi to the US dollar in 2017 was GH¢ 4.4 to US$1.
